# General practitioners’ experiences and perceptions of mild moderate depression management and factors influencing effective service delivery in rural Australian communities: a qualitative study

**DOI:** 10.1186/s13033-017-0159-x

**Published:** 2017-09-18

**Authors:** Tamishka De Silva, Anjali Prakash, Sandhya Yarlagadda, Marjia Daniella Johns, Kate Sandy, Vibeke Hansen, Sue Phelan, Sabrina Pit

**Affiliations:** 1 Westmead Hospital, Westmead, Australia; 2 Concord Hospital, Sydney, Australia; 3 Nepean Hospital, Kingswood, Australia; 4 University Centre for Rural Health, Western Sydney University, Lismore, Australia; 5 University Centre for Rural Health, Western Sydney University, University of Sydney, Lismore, NSW Australia

**Keywords:** Depression, General practice, Mental health, Mental health services, Primary healthcare, Rural health, Rural health services, Social determinants of health

## Abstract

**Background:**

Rural communities in Australia face significant disadvantages relating to geographical isolation and limited access to mental health services. Documenting general practitioners’ (GP) experiences and perception of mental health services in rural Australia may be useful to gain insight into rural GP management of mild to moderate depression.

**Aims:**

To explore GPs’ experience and views on which factors influence access to mental health services for mild to moderate depression.

**Method:**

This qualitative study was conducted in 2014 in the Northern Rivers, NSW, Australia. Data were obtained from semi-structured in-depth face-to-face interviews with ten GPs, and analyses were performed using a general inductive method of thematic analysis.

**Results:**

Most GPs believed that the current services for managing mild-moderate depression were adequate, however they also identified the need for better access and more services that were free for patients. GPs had a positive perception of management of depression in a rural setting, identifying advantages including better doctor-patient relationships, continuity of care and the proximity of services. However, GPs also identified several barriers to access to mental health services in a rural setting, including long waiting-times, inadequate patient rapport with referred professionals, cost of treatment, transportation, geographical location, stigma, and lack of education about available mental health services. As a result, GPs frequently self-managed patients in addition to referring them to other community mental health service providers where possible.

**Conclusion:**

Overall, GPs appeared relatively satisfied with the resources available in their communities but also identified numerous barriers to access and room for improvement. Rural GPs often self-managed patients in addition to referring patients to other mental health services providers. This should be taken into account when designing mental health policies, developing new services or re-designing current services in rural communities.

## Background

More than 300 million people experience depression worldwide [1]. Globally, the World Health Organisation’s (WHO) World Mental Health Surveys have revealed a high prevalence of mild-moderate depression [2]. Despite a high prevalence, there remains a substantial treatment gap in both developing [2] and developed countries [3, 4]. There is increasing evidence demonstrating the effectiveness of mental health service delivery by non-specialist health workers [3]. Indeed, in 2015, the WHO released, as part of the Mental Health Gap Action Programme (mhGAP), the second version of the *mhGAP Intervention Guide for Mental, Neurological and Substance Use Disorders in Non*-*Specialized Health Settings* [5]. There is also evidence that mental health services are best delivered in the community and at or close to peoples’ homes [4, 6]. Notwithstanding this evidence, and the ongoing call for mental health service reform globally, there remain numerous barriers that delay further mental health service reform such as reduced access to services, unequal mental health resource allocation, stigma and discrimination [6]. To combat these barriers, Abdulmalik and colleagues [6] call for a ‘balanced model of care’ that takes into account: the available resources and context; promoting a task sharing approach between hospital and community care; and increased use of community mental health services. Thus, an appropriate strategy to address the treatment gap is that GPs manage and provide interventions for mild to moderate depression.

Mental health management in rural and regional areas of Australia presents a unique challenge for GPs and other health care workers. In comparison with their urban counterparts, rural and regional communities face significant disadvantages due to barriers relating to geographical isolation and limited access to mental health services [7–10]. These barriers to mental health access may apply to various rural population groups. In particular, Hodges et al. [11] postulated that youth mental health issues may be exacerbated in rural and remote areas, due to barriers such as stigma, lack of anonymity, cost and lack of transport. A mixed method study that looked at mild moderate depression amongst older people in the Scottish highlands [12] found that GPs were more likely to prescribe medications than offering psychotherapeutic treatment. Despite this, the GPs thought that social therapeutic options were as effective as pharmacological treatment. The reason given by the GPs for not using therapeutic interventions was that they were not sure of the services available.

While previous research has explored the views of patients [13–16], mental health workers [17], and other health professionals [16], review papers by Boyd [18] and Fraser [10] have identified the need for further research relating to rural mental health issues and management. Furthermore, research conducted among rural GPs has mainly utilised a quantitative research methodology in the form of surveys [12, 19–22] with some surveys allowing GPs to leave comments. None of the surveys focussed specifically on mild-moderate depression. To our knowledge, no qualitative research has been conducted to explore rural GP perceptions of factors influencing access to mental health services for mild to moderate depression in Australia.

The true extent of disadvantage faced by rural communities in comparison with their urban counterparts is difficult to ascertain [10, 23, 24], although the evidence is rising. The validity of the universal approach to mental illness in rural communities is questioned, as there is increasing evidence that there is “inequitable distribution of mental health care services across rural, remote and metropolitan areas” [25]. These discrepancies have been attributed to patient socioeconomic status, geographical location, and adequacy and availability of mental health services [26]. Indeed, a recent study [25] demonstrated that a lower proportion of women in rural and regional areas accessed an Australian government-funded mental health initiative ‘Better Access’ Scheme compared to their non-rural counterparts, potentially reflecting a shortage of services or difficulty accessing them [25].

Therefore, the current qualitative study aims to address this health inequity issue by exploring GPs experiences and views on factors influencing access to mental health services for mild to moderate depression in rural northern New South Wales, Australia.

## Method

### Study population and recruitment

Purposeful non-probability sampling was utilised to recruit GPs for this study. A medical practitioner, affiliated with the local academic institution, contacted 27 GPs, from a variety of locations in the Northern Rivers Region, inviting interested GPs to make contact. GPs were followed up at 1 week by a phone call to the practice. Phone contact was made with a further two GPs from a coastal rural town close to the state border who both agreed to be interviewed. Finally, phone contact was made with five GPs in other communities within the area of interest, located through an Internet search.

### Data collection

Semi-structured in-depth interviews were chosen because it allowed the interviewer to use a conversation style approach, elicit discussion, and ability to probe further when required within a structured format. Additionally, GPs are time poor so the structure would allow for a more reliable coverage of all relevant topics.

Face-to-face interviews were conducted by the authors in the clinics of the participating GPs between August and September 2014. The semi-structured interview schedule, piloted with one metropolitan GP, was based on previous research and discussion within the research team [Appendix 1].

Thirty-four GPs were approached and ten agreed to participate in the study (28%). Interviews were conducted by TD, AP, SY, MJ and KS and audio recorded with two audio recording devices with participant consent. Field notes were taken during each interview. Data saturation was achieved after eight interviews upon discussion between the researchers. Verbatim transcripts were produced by the primary interviewer and de-identified prior to analysis. The transcripts were not reviewed by the participant.

### Analysis

A general inductive approach to qualitative data analysis was used in the current study. All interview transcripts were initially examined and codes were developed by the two primary interviewers (TD and AP). The developing coding list was continually modified on the basis of discussions among all members of the research team. Once agreement of the final coding hierarchy was reached, all transcripts were coded using this structure. The data allocated to each code was then reviewed, and the codes were further synthesised and grouped leading to the development of the themes. The higher level themes were closely aligned with the research questions while lower level themes arose from in vivo coding.

## Results

All consenting GPs completed the interview. There were two female GPs and eight male GPs. All worked in a permanent capacity, with two working in a solo practice and the remainder in group practices.

The thematic analyses revealed a range of important issues in the management of mild-moderate depression in rural areas. Within the broader over-arching themes of referral, barriers and enablers to management, and the adequacy of existing services, GPs raised some important local and system factors which both hindered and facilitated the effective management of mental health issues in geographically isolated areas.

### Management and referrals

#### Management

**Fig. 1:**
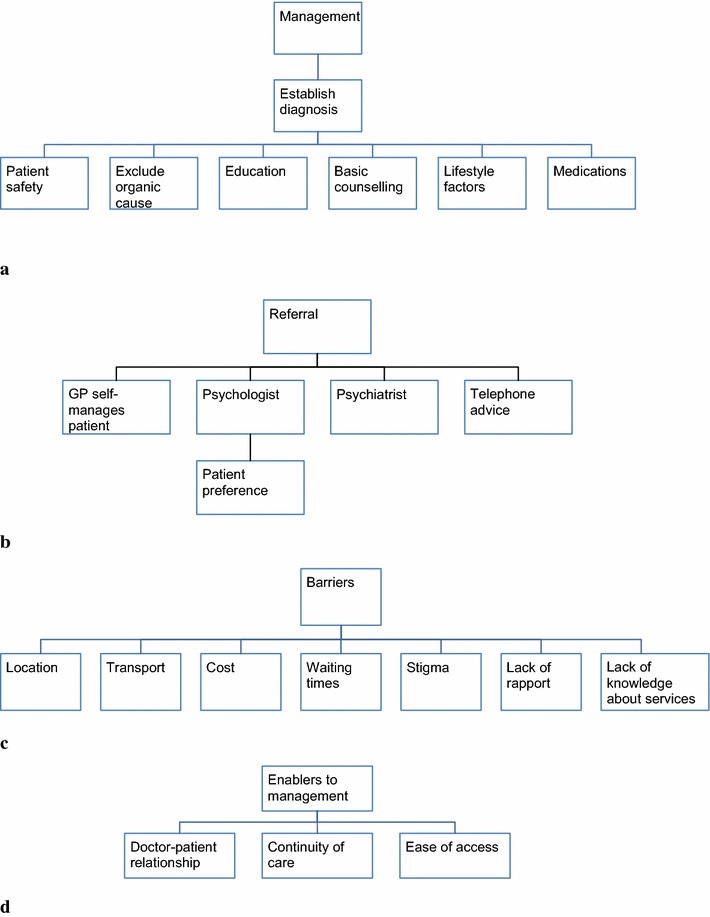
Thematic analyses of rural general practitioners’ perceptions of how factors influencing access to mental health services affects the management of mild to moderate depression. **a** Management, **b** referrals, **c** Barriers, **d** enablers

The main management methods utilised by participating GPs are presented in Fig. 1a.

Once the diagnosis of mild–moderate depression was established, and organic causes were excluded, GPs adopted a multimodal approach to manage their patients. They ensured patient safety and offered psycho-education themselves.

They offered basic counselling, prescribed medications and explored modifiable lifestyle factors that may have contributed to the patient’s depression such as sleep, exercise and alcohol intake. They also promoted non-pharmacological self-care strategies such as exercise, a balanced diet and relaxation techniques.

*If they were drinking, and self*-*medicating on alcohol or other drugs, then we might look at how to modify those… Typically they would like to exercise more but can’t for various reasons… So I might promote that by getting them to team up with friends or get involved in a local exercise program. (GP01)*

Continuity of care, encompassing the ongoing relationship and contact with their patients was perceived as essential to management and was enabled by establishing rapport and a sense of shared understanding with their patients.

Patient factors that influenced management and referral patterns included severity of depression, location, financial and employment status, ability to travel and existing co-morbidities such as drug and alcohol abuse.

A patient’s age also impacted management, influenced medication choice and their access to specialised services such as Child and Adolescent psychologists and paediatricians.

*Adolescent depression is more of a red flag so we have to be more cautious that you find the right person for them… they don’t find it as easy to talk about those issues. (GP07)*

Most GPs reported that gender, sexual orientation or cultural background did not affect management or referral patterns. However, language was at times perceived as a barrier.

*We have to use interpreter service, which is pretty cumbersome and hard to use. Sometimes those people fall through the cracks. (GP04)*

#### Referral patterns

The main referral pathways utilised are displayed in Fig. 1b.

While GPs generally managed their patients with mild–moderate depression themselves, a fundamental aspect of GPs’ management included referrals to other medical professionals, including psychologists and sometimes psychiatrists.

*I’m quite open to that idea of referring patients so they get a different person, a different reviewing process and maybe a different paradigm in psychology. (GP10)*

Psychologists were accessed through the ‘Better Access’ mental health program. These referrals were mostly guided by patient preference; GPs attempted to refer to psychologists based on patient compatibility and individual needs.

*Some psychologists are really skilled and are particularly sought after in this area* – *they’ve got particular skill sets with younger people… grief or really quite severe depression. (GP10)*

GPs reported the importance of patients being able to feel connected or being able to establish a relationship with a psychologist. In cases where rapport was not established some GPs reported they ended up managing the patient themselves.

Referral to psychiatrists were infrequent. However, they were utilised for complex cases involving co-morbidity, medication reviews or particular depression types such as post-partum.

*Sometimes when you feel that you’re getting out of your depth and you’d like a psychiatric opinion, you can get that these days. (GP06)*

When requiring psychiatrist feedback, GPs occasionally solicited telephone advice from colleagues to avoid referring to specialists with long waiting lists.

*Sometimes I might just ring a local psychiatrist to ask for suggestions and they may or may not see that person, as there could be a 3 or 4* *month waiting list, but they would give me a management plan. (GP01)*

Another reason GPs would choose to refer their patients was time constraints, which was perceived to prevent adequate management of the patients with more severe or complex problems.

*If they’ve got more significant depression that’s more in the moderate range… then I definitely refer them, because I don’t have the time. (GP09)*

### Barriers to management in a rural area

The main perceived barriers to management are shown in Fig. 1c.

#### Geographical location

A rural location was identified as a barrier; due to potential social isolation and lack of support networks experienced by some patients residing in small or isolated communities.

*There are a lot of people up here that don’t have family… if you don’t have family near then it makes having supportive family structures more difficult. (GP09)*

#### Transport

Patient’s limited ability to travel was perceived as a significant barrier, identified by nine of the ten GPs. This was due to various factors, including geographic isolation, reduced mobility, financial constraints, and lack of public transport in the area.

*There’s not really any good public transport here… if you don’t have a car it’s pretty hard, and if you can’t afford fuel it’s pretty hard, and that’s a big problem here. (GP04)*

#### Cost

Financial factors were also recognized as a barrier to access, with three GPs noting that private psychiatry fees rendered them unsuitable for some patients.

Half of the GPs noted the patient’s socioeconomic status created a barrier, whether due to unemployment or other financial difficulties. However, an equal proportion said that finances didn’t necessarily affect management and mental health specific Medicare (government-funded) item numbers alleviated these financial barriers:

*There are Medicare item numbers to help significantly subsidise their [patients’] visits to a psychologist*. *(GP07)*

#### Waiting times

Additionally, a number of GPs noted long waiting lists for psychologists or psychiatrists as an important management barrier:

*Sometimes the wait time can be… a couple of months and that can be a difficulty. (GP10)*

#### Stigma

Some GPs identified stigma as a barrier, especially for men and patients medicated for their depression.

*Mental illness has some stigma attached with it, particularly in generations in the past where any history of mental illness is regarded…as a form of weakness. (GP10).*

Stigma also plays a different role in rural communities.

*It’s difficult in a small community, someone might see them coming out of the pharmacy with medications, or coming out of the doctors surgery crying or they go to their child’s soccer match and their psychologist is there. (GP01)*

However, one GP noted the important role of some organisations in reducing stigma surrounding mental health.

*With the campaigns in place, they [Beyond Blue and the Black Dog Institute] have been really successful in bringing to the public’s attention that depression is a common thing and is readily treatable. (GP10)*

#### Lack of rapport

GPs perceived patient’s reported inability to feel connected or establish a relationship with a psychologist was found to be a barrier to management.

*I’ll make a referral to somebody and if that individual just isn’t able to establish a rapport that can be a real barrier and in those instances I find myself managing patients predominantly. (GP10)*

#### Lack of knowledge about services

One GP highlighted that they were unaware of the available services.

*With the psychologists, I get a sense that I don’t really know who is out there… There is a mental health networking group… they have a topic and particular psychologists who might present what their special interests might be, and they might present specific cases… So it would give you a sense of who’s out there. But as always we’re always too busy to fit these things in sometimes. (GP01)*

Another GP expressed the desire for more education surrounding CBT, particularly internet-based CBT:

*There is a lack of education among the GPs about how to do that [internet*-*based CBT]… I don’t really have the skills or the knowledge to know how to do it. (GP09)*

### Enablers to management in a rural area

The GPs were asked about the advantages of working in a rural environment when managing mild-moderately depressed patients and generally identified three significant factors (see Fig. 1d).

#### Doctor–patient relationship

One advantage of the rural setting was patient awareness of GPs reputation with managing mental illness. They also felt that there were opportunities for developing closer patient relationships with better knowledge of their family background.

*We are more likely to know the families and backgrounds of the people we see, as opposed to the people in the city… I can see somebody who I’d know their family circumstance, which gives you a bit of a head start*. *(GP03)*

#### Continuity of care

Another benefit was that the rural setting allowed for continuity of care, which is the ongoing relationship, and management of a patient over time.

*There’s a lot more continuity of care and follow*-*up… so there’s no people falling through the gaps in that way. (GP10)*

Three GP’s alluded to the stronger support systems in their rural environment fostered by the patient’s family and the close-knit community. However, as discussed under barriers, sometimes the opposite was true in cases where no family was living close by to be able to support people with mild-moderate depression.

<*Village name*> *is quite a close community, people seem to keep an eye on one another and seem to know one another*. *(GP07)*

#### Accessibility of services

Two GPs identified the relative accessibility to two major cities, as a facilitator in the management of mild–moderate depression in the rural area.

When compared to the city, four GP’s noted that the rural environment provided convenience and proximity to services:

*The Northern Rivers area has more psychologists and psychiatrists than Sydney’s Northern Beaches suburbs*. (*GP08*)

The same GP also acknowledged the longer waiting lists in the city, while another GP discussed the advantages associated with practicing in a region identified as an area of mental health need, leading to new initiatives such as the New Access program.

### Adequacy and availability of services

There are many services that GPs believe assisted with their management. Despite the barriers mentioned above, nearly all GPs thought that current systems and services for mild–moderate depression were relatively adequate. However, most GPs noted at least one service that could be improved.

#### Psychologists

Most GPs felt that psychology services in the area were satisfactory.

*We’ve got a lot of psychologists that we can refer to… I think generally the services are pretty good. (GP04)*

However some GPs indicated the need for better access to psychologists, particularly government-funded, as they played an important role in the management of mild-moderate depression and could potentially avoid the use of medications and medical involvement.

*The better availability of psychologists and counselors does help, because… with people with mild depression, it can be treated without medication, without extensive medical involvement, and just having someone literally to chat to. (GP03)*

Many GPs felt that the Medicare Local Mental Health Service, which provided free public psychologists in the main regional town, was a good and valuable service. However, most GPs also mentioned that the service was heavily utilised due to being a free government-funded service.

#### Psychiatrists

Most GPs indicated that having more psychiatrists in the area, particularly free government-funded psychiatrists, would be beneficial; however many acknowledged that they were more useful in managing severe depression, than mild-moderate.

*Not many of the psychiatrists bulk*-*bill* <*government funded*> ***…*** *I don’t often do that for mild*-*moderate depression but if things aren’t working and it’s becoming more severe, then that can be a barrier*. *(GP 09)*

#### Acute care service (ACS)

A number of GPs noted the ACS as a management option for severe depression. The ACS forms part of locally delivered government-funded Community-based Mental Health Services. However, some felt that underfunding limited the ACS’s ability to manage patients with mild–moderate depression.

*What we’re talking about here is mild*-*moderate depression so that’s really just a very small part of what they [the ACS] do, and because of the budgetary constraints they really aren’t able to provide much of a service for people suffering with that… I would like to see them [the service] better resourced. So that rather than focusing upon people with psychotic illnesses, they’ve got more scope to deal with people with depression. (GP10)*

#### Community-based services

Two GPs noted the availability of mental health professionals in their area that make home visits and see patients in local community centres. In one town, the local mental health professional (LMHP) was perceived to provide a useful service.

*[The LMHP] sees patients down at the community centre… [their] aim is really a liaison between the community and the psychiatric services… will sometimes see them as a primary provider before I get to see them. (GP04)*

However, one GP thought that community services could still be improved by allowing LMHPs to conduct home visits with people with mild-moderate depression.

*I guess some sort of outreach service would be fantastic, and if mental health services had mental health professionals that could actually go to people’s houses and see them in that context and prescribe for them and manage them as a unit, that would be really good. They do that for the more serious mental issues, but for the milder ones that’s really not available. (GP04)*

#### Drug and alcohol services

While one GP thought that drug and alcohol counselling was a good service, another GP suggested better coordination between mental health services and public drug and alcohol services.

*Heaps of people in this area are you know, smoking too much pot, drinking too much alcohol, as part of their illness and there’s no dedicated public alcohol and other drugs services. There’s [a drug and alcohol treatment centre in the main regional town] which will do an inpatient detox, and that’s it. And the AOD *<*Alcohol and Other Drug*>* space and the mental health space really need to be working together as a team. So the sort of traditional response you get from some of the mental health services is “we won’t look at this patient while they’re on drugs” which is just stupid because unless you treat the mental health you’re not going to get off the drugs, you have to treat both at the same time. So a coordinated team approach would be really good. (GP01)*

#### Other services

While, one GP noted a definite lack of adolescent mental health services, another perceived a new counselling service ‘NewAccess’ (http://www.beyondblue.org.au/get-support/newaccess), to be beneficial.

*Mostly they will do it by telephone access but sometimes they get the person to come in and the results seem to be quite good. I have sent a few people there… It’s made a big difference. (GP08)*

## Discussion

Overall, GPs were relatively satisfied with the resources in their communities, but also identified barriers to management unique to their rural location. The most salient barriers included stigma, GPs’ time constraints, geographical location, referral options, patient transport, cost, adequacy and availability of mental health services. Whilst some of these barriers may be applicable to both urban and rural GPs, some barriers have a different meaning in the rural context. For example, GPs perceive that stigma, as a barrier in a small rural town, plays a different role to accessing mental health services than in the city as patients are more likely to be recognized by others and more likely to bump into their mental health service providers in a rural community. Similarly, public transport can be more difficult to access in rural areas, which reduces patients’ ability to access local mental health services providers.

These barriers are in accordance with recent literature that suggests an inequitable delivery of government-subsidised mental health services through the Better Access initiative [26]. It is more difficult for the socioeconomically disadvantaged patients to access specialist care [10] and both disadvantaged and rural areas typically have limited services available, potentially provided by less highly trained providers [10]. A relatively new Australian initiative, NewAccess, is an early intervention program for people with mild to moderate anxiety and depression. It is free for the patient and includes Low Intensity CBT. The services also link patients to local community networks and service providers such as housing and employment [27]. This free service may assist in further improving access to mental health services for mild moderate depression in rural areas. Indeed, one GP in this study mentioned that this program had made *‘a big difference’.* The initiative has currently been rolled out in four areas in Australia [28]. Lack of knowledge of local health professionals were also mentioned by GPs. GPs also expressed the desire that they would like more training and education to inform them of new existing services, including internet-based programs. The implementation of HealthPathways in Australia and New Zealand will partly address this issue (http://www.healthpathwayscommunity.org). HealthPathways is an online tool and local health pathways are developed by local clinicians to assist and inform local primary care providers on assessment, management, referral pathways and services available locally for specific conditions such as mental health.

Many GPs also perceived enablers in managing mild-moderate depression in a rural setting, including improved doctor-patient relationships, continuity of care and ease of access to particular services. The latter enabler appears to be contradictory with other findings and needs to be put into context. It is likely that GPs who were familiar with new mental health services and those who live in an affluent coastal area perceive to have better access than those who are not familiar with the services or live in less affluent areas. Indeed, there were significant discrepancies between the barriers identified by each GP in their areas. Literature has deemed stigma a barrier to mental health care [7, 8, 10, 11], and whilst some GPs agreed with this, others also stated that public mental health campaigns were effective in reducing stigma. Furthermore, whilst all but one GP stated that transport was a barrier to management, nearly half the GPs agreed that the rural location enabled convenient access to services. Similarly, whilst some GPs experienced difficulties accessing free government—funded psychiatrists and psychologists, about half agreed that the mental health resources in their area were adequate in both quality and volume to manage patients with mild–moderate depression.

These discrepancies suggest a disparity between the needs of varying communities in the Northern Rivers region, a potential treatment gap [2, 6] and potential inequalities in the distribution of mental health resources [6]. This raises questions about the validity of the universal application of mental health policies to the rural population. Rural communities are often presented homogenously, characterised as “isolated and impoverished, exposed to stressful environmental hazards and ‘a culture of violence” [10]. This perspective fails to address the vast diversity between individual rural communities and their needs. Increased understanding of the mechanisms and development of local initiatives that contribute to improved mental health outcomes in different rural areas will promote further development of effective policies.

### Study strengths and limitations

To our knowledge, there is limited literature that has used qualitative methodologies to understand GPs view on management of mild–moderate depression in the rural setting. In this study, we directly engaged with local GPs in the setting of their practices. The semi-structured interview and qualitative design (Appendix 1) enabled a focused exploration of this topic, and the data was further validated through discussion with a local mental health expert.

Whilst a qualitative approach allowed for a greater exploration of the mental health resources, a few factors may have influenced the results. A small sample size may have influenced some responses. However, data saturation was achieved, as the latter interviews yielded little new information. The participating GPs were predominantly from larger rural towns and this may have limited our exploration of resources in more isolated remote areas. Generalisability of the study’s findings to other geographical areas need to take into account local variations. With the predominant female to male ratio (1:4) in the current study, this potential gender bias should be acknowledged as female GPs are known to manage mental health patients differently from their male counterparts [29].

### Implications for practice

The implications for practice and policy from rural GPs perspective include more free government-funded psychology and psychiatry services and extension of the of Community-based Mental Health Services such as the Acute Care Service, increasing home visits [4, 6] and assessment, and enabling more consults. In addition, improving adolescent mental health services and a stronger liaison between mental health and drug and alcohol services was perceived as beneficial. Increased GP education about available resources will also aid management of mild/moderate depression. Finally, increased financial support, such as expanded government-funded items or mental health education grants and improved access to transport and community services for patients with mild to moderate depression, would also assist rural GPs in improving mental health service delivery.

## Conclusion

Overall, GPs appeared relatively satisfied with the resources available in their communities but also identified numerous barriers to access. These discrepancies suggest a disparity between the needs of varying communities within rural areas. Rural GPs self-managed patients but also referred patients to other mental health services providers where possible. GPs believed that their management of mild-moderate depression is facilitated by appropriate services but there is room for improvement to ensure effective mental health service delivery in rural Australian communities. This should be taken into account when designing mental health policies, developing new services or re-designing current services in rural communities.

## Appendix 1: Script summary

- Patient demographics.
- Management of patients with mild-moderate depression.
- Barriers to services and adequacy of services in the area they live in.
- Main advantages and disadvantages of managing patients in rural areas.
- Additional comments.
